# Goals and Principles of Providers Working with People Experiencing Homelessness: A Comparison Between Housing First and Traditional Staircase Services in Eight European Countries

**DOI:** 10.3390/ijerph16091590

**Published:** 2019-05-07

**Authors:** Marta Gaboardi, Michela Lenzi, Francesca Disperati, Massimo Santinello, Alessio Vieno, Aurélie Tinland, Maria J. Vargas-Moniz, Freek Spinnewijn, Branagh R. O’Shaughnessy, Judith R. Wolf, Anna Bokszczanin, Roberto Bernad, Ulla Beijer, José Ornelas, Marybeth Shinn, HOME-EU Consortium Study Group

**Affiliations:** 1 Department of Developmental and Social Psychology, University of Padova, 35131 Padua, Italy; michela.lenzi@unipd.it (M.L.); francesca.disperati@unipd.it (F.D.); massimo.santinello@unipd.it (M.S.); alessio.vieno@unipd.it (A.V.); 2 Department of Research and Innovation, Support Unit for clinical research and economic evaluation, Assistance Publique–Hôpitaux de Marseille, 13385 Marseille, France; aurelie.tinland@gmail.com; 3 APPsyCI—Applied Psychology Research Center Capabilities and Inclusion, ISPA-Instituto Universitário, Lisboa 1149-041, Portugal; mariajvargasmoniz@gmail.com (M.J.V.-M.), jornelas@ispa.pt (J.O.); 4 FEANTSA, European Federation of National Organisations Working with the Homeless, Bruxelles 1210, Belgique; freek.spinnewijn@feantsa.org; 5 Department of Psychology, University of Limerick, Limerick V94 T9PX, Ireland; branagh.oshaughnessy@ul.ie; 6 Impuls-Netherlands Center for Social Care Research, Radboud Institute for Health Sciences, Radboud University Medical Center, Nijmegen 6525 EZ, The Netherlands; judith.wolf@radboudumc.nl; 7 Institute of Psychology, Opole University, Opole 45-052, Poland; abok@uni.opole.pl; 8 Rais Fundación, 28028 Madrid, Spain; roberto.bernad@raisfundacion.org; 9 STAD, Stockholm Center for Psychiatry Research and Education, Karolinska Institutet, Stockholm 113 64, Sweden; ulla.beijer@ki.se; 10 Department of Human and Organizational Development, Peabody College, Vanderbilt University, Nashville, TN 37203, USA; beth.shinn@vanderbilt.edu

**Keywords:** goals, principles, service delivery, housing first, providers, homelessness, cross-national study

## Abstract

The implementation and adaptation of the Housing First (HF) model represented profound changes the structure and delivery, goals, and principles of homeless services. These features of homeless services directly influence providers, their work performance and the clients’ outcomes. The present research, conducted in eight European countries, investigated how social providers working in HF or TS (Traditional Staircase) describe and conceptualize the goals and the principles of their services. Data were collected through 29 focus group discussions involving 121 providers. The results showed that HF and TS had similar and different goals for their clients in the following areas: support, social integration, satisfaction of needs, housing, and well-being. HF providers emphasized clients’ autonomy and ability to determine their personal goals, with housing being considered a start on the path of recovery, while TS were more focused on individual clients’ basic needs with respect to food, health and finding temporary accommodations. HF providers privileged the person-centered approach and housing as a right, while TS providers were more focused on helping everyone. Implications of the results are discussed as suggestions both for practice and for research.

## 1. Introduction

In Europe, the Housing First model (HF) of homeless services delivery [1,2,3] is gaining traction as an alternative to the Traditional Staircase model (TS). In HF, people in homeless situations move directly from the street into independent accommodation with wraparound supports, and without any pre-condition to start a process of recovery [4]. Unlike HF, TS programs typically require treatment and sobriety, with the goal of stabilizing people prior to providing housing.

Previous studies have shown the effectiveness of the HF model on people experiencing homelessness’ outcomes (recovery, housing stability) [5,6,7,8], but less research has taken place in Europe [2,9,10,11] or focused on the providers’ point of view [12,13,14]. However, in Europe, the Program ‘Europe 2020′ emphasizes the need to develop integrated strategies to reduce social exclusion and extreme marginalization. Horizon 2020 finances the European project HOME_EU: ‘Homelessness as Unfairness’ (2016–2019), which aims to provide a comprehensive understanding of how European citizens perceive, tolerate, and contest homelessness, through the integration of multiple perspectives such as that of citizens, policy makers, people experiencing homelessness, and social providers. The HOME_EU consortium is composed of 12 partners in 9 European countries (Belgium, France, Ireland, Italy, the Netherlands, Poland, Portugal, Spain, and Sweden). This study of service providers’ experiences working in either Housing First or traditional services about their goals and principles is part of project HOME_EU.

In particular, this study focused on the goals and principles adopted by homeless services providers from HF and TS services. The HF model introduced a paradigm shift in the service system [15], due to radical change in basic principles guiding work with homelessness. Tsemberis [4] developed the original HF model (Pathways to Housing), which operates according to five key principles: (a) Housing: immediate access to housing without conditions; (b) Choice: participants’ free choice about where and how they live; (c) Harm reduction philosophy: separation of housing from therapeutic treatment; (d) Support: an individualized and person-centered approach that aims to support the person in his/her process of recovery; (e) Social integration: support that seeks to integrate the people in the community where they live. In the Housing First Europe Guide [16] for the implementation of HF in Europe, the eight principles are: housing is a human right; harm reduction; choice and control for service users; active engagement without coercion; separation of housing and treatment; person-centered planning; recovery orientation; flexible support for as long as is required. In contrast, TS services operate according to the following principles: people with experiences of homelessness must learn to live stably in their own homes; treatment and medication for physical and mental health problems are prerequisites for access to housing; anti-social behavior should be prevented and minimized; and, abstinence and sobriety are required before service users can access supports and housing [16].

Compared to traditional services for the homeless, the principles of HF involve not just a change of practices, but a change in the perception of people experiencing homelessness, the provider–client relationship, and the social system. Padgett, Henwood, and Tsemberis articulate: “HF is a whole-system approach. HF portends changes that are systemic, not just programmatic” [17] (p. 167).

The changes in system delivery introduced by the HF model depend on several factors. For example, Nelson et al. [15], found two major processes in the implementation of HF in six Canadian communities: (a) changes in system capacity and (b) changes in the coordination of system elements and collaboration among system stakeholders. Regarding the changes in system capacity, at the individual level, the participants (stakeholders) noted that the shift to the HF model involved a change in the mindset of service delivery stakeholders (e.g., belief in consumer choice and recovery). Also, they found that this change was not always shared with stakeholders outside HF program. In fact, at the organizational level, they recommended that before any system change occurs, dialogue should take place among community stakeholders, whereby a consensus on HF’s value is gradually solidified.

Henwood, Shinn, Tsemberis, and Padgett [13] showed that the perspectives of frontline services providers in HF and TS services are important aspects of the paradigm shift in favor of the HF approach. HF providers reported greater endorsement of consumer values such as the right to refuse treatment, less endorsement of systems values such as a requirement to be clean and sober prior to living independently, and greater tolerance for deviant behavior than TS providers. Their findings indicate that differences in philosophy and structure directly influenced providers and their work. For example, providers in both types of programs considered housing important; however, providers in TS services were pre-occupied with housing services users, whereas HF providers were able to focus more on clinical issues, since their services users had already obtained housing [18].

Although the aforementioned studies showed that a change in social providers’ goals and principles usually characterizes services adopting the HF model, the shift to new goals and principles is not an automatic or easy process for providers. Studies have shown that the level of congruence between HF values and the personal values of the staff has an impact on program fidelity: higher fidelity when staff members’ values aligned with the HF values, lower fidelity when the staff adopted a quid pro quo or transactional approach to working with clients [19,20,21]. Manning and Greenwood [12] studied the influence of service providers’ values on service user’s recovery and found that compared to interventions guided by provider-led values, interventions guided by consumer-led values (i.e., a key HF principle) resulted in better recovery experiences for homeless service users.

Thus, it is important that the basic principles of the HF model are not lost in the implementation process. This is tricky, because during the implementation process, local adaptations to the HF model can result in modifications to core principles. Research has shown that fidelity to a model (and principles) helps in achieving positive outcomes [22], and fidelity helps to ensure that the principles of the new model are adopted. Macnaughton et al. [23] used a mixed methods approach to study the factors which influence fidelity to the HF model. Their findings indicate the importance of value congruence between staff members and the HF model, and the importance of organizational culture for program fidelity, which in turn leads to positive outcomes. In particular, staff members’ commitment to the project and recognition of its value, despite the difficult nature of the work, provided a base for the growing expertise of the team. Personal values are difficult to influence through training, so building staff capacity starts with the recruitment of staff with values aligned with that of the project. More recently, a multi-country HF fidelity study was conducted in 10 different HF programs [24]. The authors indicated that important organizational facilitators of HF fidelity were regular training and team building centered on HF principles, and organizational and staff commitment to HF values.

Widely shared goals and principles, and well-defined work procedures, are crucial for successful program implementation, including the program’s effectiveness, the quality of clients’ and providers’ relationships, and everyone’s well-being. Findings from across several studies indicate that strong performance is associated with well-defined team goals, regular feedback on performance, and clear guidelines for coordinating the team’s work [14]. These factors could be a way to promote empowerment and well-being among an organization’s members (both clients and workers). Without clear and realizable goals, providers could experience feelings of powerlessness, depression, and fatigue. Over time, unrealistically lofty goals and high expectations my increase disappointment and burn out [25,26,27].

Given the importance of goals and principles to the development and maintenance of effective organizations, Maton [28] included these components in his proposed empowering community settings model, which served as the theoretical framework for the current research. Based on his review of the literature, Maton proposed a set of six organizational characteristics that promote organizational empowerment: a setting’s group-based belief system, core activities, relational environment, opportunity role structure, leadership, and mechanisms for setting maintenance and change (i.e., the organizational mechanisms used to adapt both to internal and external changes). Each organizational characteristic is associated with psychological mediators that, in turn, are associated with higher levels of empowerment for setting members. In particular, we focus our work on the component ‘belief system’, which refers to the setting’s ideology and values. According to the model, an empowering organization should have: a well-defined and shared belief system, i.e., the group members should have a shared vision and a larger purpose; developed to inspire change, with salient goals and clear means, and characterized by a strengths-based approach, considering the members as resources with capabilities. This kind of group belief system can contribute to empowerment by promoting higher levels of awareness and motivation among its members. Also, the belief system is an integral part of setting culture because beliefs guide patterns of behavior, with norms and practices, to produce desired outcomes. Then, it might be particularly important for improving clients’ outcomes, especially for those working with people with complex needs. Moreover, Maton [28] observed that, when all six sets of empowering organizational characteristics are in place, community-based settings function as vital relational communities with a triple effect on: increasing numbers of empowered citizens; empowering individual members’ radiating influence; and impacting on external organizational activities. The potential impact of empowering organizations is not limited to inside the setting (members) but also on the community. For these reasons, Maton’s framework has been used as a tool to assess empowering processes across ecological levels in community organizations (e.g., [29]). For the first time, we used this framework to analyze the homelessness services, considering the importance of both the individual level (increasing clients’ outcomes) and the systemic level (changing of the service delivery).

Considering the importance of goals and principles in the implementation and adaptation of a new model of services for people experiencing homelessness, and the lack of studies analyzing the point of view of social providers in the field, we aimed to understand how social providers in eight European countries, working in HF or TS, describe and conceptualize the goals and the principles of their services. More specifically, we aimed to: (a) to explore the goals and principles of social providers in HF and TS services in eight European countries; (b) to compare HF and TS providers’ goals and principles across the countries.

## 2. Methods

### 2.1. Procedure

The study was conducted in eight European countries involved in the HOME_EU project (France, Ireland, Italy, the Netherlands, Poland, Portugal, Spain, Sweden). A research protocol was shared among partners and approved by the European Ethics Committee (Ref. Ares (2017)535021-31/01/2017) and the Ethics Committee of each University/Research partner of the consortium.

The data were collected through focus group (FGs) discussions during May–June 2017. Participants were recruited through convenience sampling within HF and TS programs. To be eligible, service providers must have had at least six months of experience in their role with the service. At minimum, four service providers were recruited per FG.

Before the FG session, consent forms were provided to each participant, adapted on the basis of the laws of their country. Local researchers in each country conducted all FGs, which were audio-recorded and lasted approximately 60–90 min.

The FGs started by researchers asking participants to describe the main aims of their program. Afterwards, specific questions explored the six organizational characteristics (belief system, core activities, relational environment, roles, leadership, and mechanisms for setting maintenance and change) of empowering community settings model [28]. In this study, we started by analyzing the responses regarding goals and principles (belief system), and considered them as bases of the other organizational characteristics.

### 2.2. Participants

In total, 29 focus groups (15 HF, 14 TS) were conducted with 121 participants, 70 female, and 59 male. All participants had at least six months of experience in the service. Two HF and two TS focus groups were conducted in each country, with the exception of Ireland, where one additional HF FG was conducted. Only two TS FGs were conducted in Poland, because there were no HF programs there at the time.

### 2.3. Data Analysis

We used thematic analysis [30] to interpret responses to questions following two steps:

Step 1—Country analysis: all the focus groups were transcribed verbatim in the local language. Two independent coders conducted the qualitative coding in each country. Each coder independently read the data for familiarization. Next, each coder created a first set of codes based on the empowering community settings model [28], selecting all complete responses (by eliminating potential sentences not completed or without a meaning). Each partner created a Microsoft Word document (translated into English) that contained listed excerpts from transcripts that related to service goals and the category ‘belief system’ of the empowering community settings model.

Step 2—Cross-national analysis: The second step involved coding the data (the answers referred to goals and principles) through a thematic analysis [30]. Two independent researchers developed codes regarding goals and principles and compared them to create a final coding framework. Responses were coded into the resulting categories irrespective of the type of service or the country. Categories were compared and coders discussed discrepancies until they reached agreement. Finally, we examined in which services (HF and TS) and countries each of the categories emerged.

## 3. Results

We identified two main themes: goals and principles.

### 3.1. Goals in Homelessness Services

Regarding the goals of their services, providers talked about different aims relating to the clients. The main goals that emerged from providers of both kinds of services are shown in Table 1.

Providing support to clients is considered an important aim among providers. In their view, support should be tailored to clients’ needs. According to some providers, clients should have a say in deciding their goals, while according to others, clients need providers’ support to achieve goals chosen by professionals. For example, service providers may help clients connect to local services needed for their recovery. Also, according to providers involved in the study, the support should facilitate client autonomy, i.e., the ability to live independently in the house and in the society. Another goal of the services delivery is social integration, which entails (re)-activation of formal and informal social networks, as well as general integration within the society/community as citizens.

In relation to clients’ outcomes, other goals mentioned by providers were satisfying clients’ basic needs, by providing food, showers, and clothes and protecting the clients’ safety by helping them finding a temporary accommodation or a house. Providers also aimed to help clients improve their well-being, in terms of physical and psychological health. Another goal mentioned during the focus groups is helping clients to find a job or an activity, in order to have sufficient resources to live independently or to have something to do during the day and be recognized and appreciated in society.

### 3.2. Principles in Homelessness Services

Service providers (both in HF and TS services) identified several principles guiding their work, as summarized in Table 2. They underlined the importance of respecting clients and acknowledging their dignity and humanity to make them feel accepted and welcomed. They explained how working with marginalized and stigmatized people makes it even more critical to show them respect and treat them with dignity. Another principle is helping everyone unconditionally, which refers to supporting the clients despite their difficult situation and what happened in their past. The providers also emphasized with the importance of considering the person at the center of the support, by adopting an individualized approach and considering clients as active agents in their projects. Another principle mentioned by participants was that housing is a right for everybody. Some providers described how the general principle of social justice guides their work, supported by the belief that all people deserve social improvement, regardless of their life condition.

The providers in the FGs, both in HF and in TS, also discussed some organizational factors that helped or hindered their application of their principles, as summarized in Table 3.

In particular, they considered difficulty in putting the principles into practice, due to organizational limitations (e.g., lack of resources, workload, difficulty in communication). For example, they reported that their workload does not always allow enough time to nurture relationships with clients. At the same time, the providers underlined the importance of having a mission that guides their daily work, sharing values with colleagues, and having a united team. Sometimes the providers had difficulty sharing and adopting the organization’s principles because of the lack of a good system of communication within the organization. Also, providers identified the multidisciplinary approach as a key ingredient helping put principles into practice for two main reasons: first, having different people in the team allows them to share the responsibility of supporting people with complex needs (e.g., more providers support a client and this facilitates a person-centered approach); second, having people with different professional skills and background allows the adoption of a broader approach in addressing problems.

Finally, the results show the importance of sharing the principles of the services with partners outside the team, in order to align their work with other homelessness services and to create a consistent system change in the network of services for people experiencing homelessness.

### 3.3. Are there any Differences in Goals and Principles between HF and TS in Eight European Countries?

Most of the goals and the principles that emerged in the study are common to the two types of services (HF and TS), but providers discussed them differently. As shown in Table 4 and Table 5, each goal and principle has sub-themes that occur differentially in HF and TS. The tables show the number of services in which each goal (and sub-theme) or principle was mentioned at least once.

Regarding goals, HF providers emphasized client autonomy, i.e., living independently in society: “first of all, the goal is to support them in a path of autonomy” (Italy, HF2); “that thanks to us, they fly with their own wings in our society” (France, HF1). They also stressed that individual clients should decide their own goals: “the person decides by him/herself, in autonomous way, what he/she wants or does not want” (Spain, HF2).

In TS services, the focus is still on individualized support, but it is focused on clients’ basic needs (food, shower, shelter) and problems as seen by the service provider. As a participant noted: “the final aim of the professionals is to provide support to the user and to her/his situation, that can be very diverse and also imply different areas” (Spain, TS2); “we diagnose the problem, we diagnose specified life difficulty, such a certain range of the problem, we set a goal, the most important action and let’s go, of course according to certain plan” (Poland, TS1). In both kind of services, providers pointed up the importance of clients’ integration in the community, without any significant differences in the two services.

Satisfaction of basic needs is a goal described by participants from both HF and TS. Providers from TS focused especially on clients’ safety. One said their goal was “to offer a level of safety, because they come from the streets” (the Netherlands, TS1); “in the evening we try to maintain a positive and safe climate in the shelter” (Italy, TS2). Also, the goal of housing was described differently by participants from the two services: HF providers declared the importance of providing housing, defined as a human right and a first step in the path to recovery: “it is all about basic needs and fundamental rights, such as having your own home” (Sweden, HF1). In contrast, TS providers described housing as a future goal, as a final step of a process of becoming housing-ready, for example: “that will hopefully lead to their own residence in the end” (Sweden, TS2).

In relation to clients’ well-being, providers in TS services more frequently referred to clients’ physical health as a need, e.g., “it is true that, initially, my work was to provide shelter, whereas now the health problems are really important” (France, TS2). In HF, providers focused on general well-being and recovery as broader goals, to “allow people to get off the street if they wish, and to start on a path to recovery” (France, HF2).

Finally, the goal to help people to find a job or an activity was mentioned only in TS services. For example, one provider from Spain hoped to “promote and to facilitate the access to formative resources, labor training directed to facilitating the social insertion of the users” (Spain, TS1); and another in Italy said their goal was “not only the house, they need to work but the job is a difficult for everyone, however, it is to incorporate them into society, making them feel useful and appreciated” (Italy, TS2).

Regarding principles, providers in both types of services emphasized the importance of social justice, respect, and dignity. In HF, the focus was on maintaining a person-centered approach, “the intervention is very individualized, that is, being very person-oriented and being appropriate to the person with whom we are dealing” (Portugal, HF1); “vision: client central and responsible. In that sense, Housing First fits” (the Netherlands, HF1); “it is important the involvement of the person” (Italy, HF1). In TS, services the providers highlighted the importance of helping everyone unconditionally: “despite everything that happened in their past, these clients are welcome here. I think that is wonderful” (the Netherlands, TS1). Housing First providers emphasized unconditional acceptance: “no matter how many problems you have, you will be helped” (the Netherlands, HF2). Finally, the principle of housing as a human right was mentioned only by providers of HF services: “the house is a right and the house is where you will be, independently of how you decide to live” (Spain, HF1), “we wouldn’t be here if we didn’t have a strong belief that someone deserves a home” (Ireland, HF2).

Both HF and TS providers noted that it is often difficult to put principles into practice because of organizational challenges like lack of resources, workload, and pressure from line managers. As one participant noted: “I understand that we put the persons at the center, but on the other hand it is just what is written. On our daily work we find some pressures that make the service leave people aside” (Spain, HF2); “when you have a 100–115 cases workload […] you cannot support each person as a professional as he/she deserves” (Spain, TS2); or “if a client comes in and starts with Housing First, the organization ideally would like the coach/supervisor to stay with the client, but this person has no Housing First background or training” (the Netherlands, HF1); “the organization has a vision that they are there for everybody and that they are not selective in attracting certain clients just for financial benefit. Maybe, this is the main problem: the financial aspect. But this organization is indeed available for everybody” (the Netherlands, TS1).

Nevertheless, in HF services, there was a stronger emphasis on the importance a clear mission to guide their daily work: “if you believe what you are doing is right, and you are supporting that service user as best you can, if you believe you’re going to see it through with them, that’s what gets me through” (Ireland, HF2); of sharing values and having a united team: “we can work very differently but I think our vision is always the same. We all have a common goal and a common mission” (Sweden, HF1).

In HF services, providers emphasized benefits of having a multidisciplinary team for putting principles into practice: “I find that multi-referencing [flexible and multidisciplinary] is a good way to make the person central to the support that he is given” (France, HF2). They also highlighted the importance of sharing the principles of the services with the local services or authorities outside the team: “maybe other services don’t have the same values, […] but if the team was all the same, if the people who provide accommodation were all on the one page, it would be different” (Ireland, HF2). This aspect is connected to the aim to create systems change in services for people experiencing homelessness: “linking practice and politics and trying to somehow move between how the practice of social intervention from a more innovative perspective can contribute to changing social policies” (Portugal, HF1).

## 4. Discussion

This research aimed to understand how social providers, working in HF or TS services, describe and conceptualize the goals and the principles of their services, exploring the similarities and differences between the two models in eight European countries.

Connecting our result with Maton’s framework (guiding our focus group questions), some themes reflect characteristics of the group-belief system [28]. To be empowering, services need a shared belief system that inspires change, is strengths-based, and focused beyond the self. To inspire change, the service should have salient goals and a clear means to achieve them. To be strengths-based, the service should consider members (workers and clients) as resources with capabilities: the principles of dignity, respect, and the person-centered approach are consistent with this aspect, especially in HF. Finally, to be focused beyond the self, the member of a service should have a shared vision and a larger purpose: shared vision was emphasized more by HF providers, but larger purposes (e.g., support people to get out of homelessness) were mentioned in both kind of services.

In general, regardless of the kind of service they worked in, providers indicated that their main goal was to support clients with social integration, basic needs (food, shower, health), housing requirements, and well-being. In relation to principles, the providers in both HF and TS services emphasized the importance of a person-centered approach based on dignity, respect, and humanity; they also identified social justice and unconditional acceptance as key principles that underpinned their practice.

Providers in both types of services sought to improve clients’ community integration. This result is in line with the literature because findings on the impact of services on community integration have been mixed and without clear differences between the two models [31]. However, in line with findings by Henwood, Shinn, Tsemberis, and Padgett [13], there were some differences between providers in HF and TS services in terms of their goals and principles. HF providers emphasized the importance of supporting clients’ autonomy and choice over their own personal goals, with a focus on providing a house as a start on the path to recovery and engagement. In relation to principles, the providers in the HF services favored the person-centered approach and believed housing is a human right. Providers in HF emphasized all the principles of the original HF model [4]: housing, client’s choice, support, integration; and also the principles of the HF guide in Europe [16]: engagement of the clients, person-centered planning; recovery orientation; flexible support. Although harm reduction and the separation of housing and treatment were not mentioned, HF providers discussed the need to help everyone unconditionally, and so implied that there were no requirements to access the program. The most common goal identified is giving a house to the clients, mentioned in most HF services. As shown by Manning and Greenwood [12], a major focus on clients’ choice and rights, using a person-centered approach with interventions guided by consumer-led values, has the potential to improve clients’ recovery outcomes.

TS providers were more focused on clients’ basic needs than housing: finding a temporary accommodation, taking care of health problems, promoting safety, helping clients to find a job/activity. The implicit assumption was that clients need professionals to make decisions for them regarding their needs in order to become ‘housing ready’. Their support is personalized, but clients are not involved in choosing their goals. Housing is seen as a distant and uncertain goal, not an urgent, immediate goal or a right [32]. This exclusive focus on basic needs put TS clients at risk of getting caught in the ‘institutional circuit’ of streets, homeless services, hospitals and jails [33], in which homelessness is managed, but not ended [34]. TS providers’ descriptions of their work did reflect principles of respect for clients and a person-centered approach, but they focus less on working to establish a strong relationship with the clients than on meeting their basic needs.

The person-centered approach was the only principle discussed by providers in both types of services, although to a greater extent by HF providers. It is possible that the main difference between the services, more so than in their basic principles, lies in the structure of the services. For example, only TS providers described the importance of finding a job or economic resources for clients, and this might be because clients need more resources outside the program to exit from homelessness (and find housing), while in HF programs housing is the starting point.

The influence of organizational factors on providers’ principles was expressed by providers of both kinds of services (but only in four countries), especially in relation to organizational constraints like workload and lack of resources. For example, providers in HF services emphasized the importance of having a multidisciplinary and flexible team, sharing values among the team (mentioned in seven countries but especially in HF services), and having a clear mission. In fact, the literature showed that teams with well-defined goals [14] perform better. Damschroeder et al. [35] noted that, in addition to good professional skills, strong congruence between staff, and program principles is important. Congruence of principles is important for HF programs because of the profound change in values that is required of providers who move from TS to HF. In fact, in our focus groups and interviews, providers underscored the importance of sharing principles outside the team to create innovation. For this reason, Nelson et al. [15] highlighted that dialogue among community stakeholders, during which a consensus on HF’s values can be gradually solidified, should precede the implementation of HF programs.

In light of these results, we offer some recommendations for research and practice. First, future research should investigate which dimensions are involved in a relation of support between provider and client and how the providers work to support the clients. Second, future research should focus on which factors create the working conditions that support the workforce in this field, thus helping to reduce the gap between principles and practice. In particular, research should identify the obstacles that prevent the translation of principles into practice and explain how they affect how service providers work with people in homelessness. Third, the different sociopolitical environments that facilitate or exert more pressure on program implementation should be examined [36]. Future research could examine potential differences in goals and principles between services and across countries, for example in welfare, type of organization, number of employees, provider–client rapport, style of leadership, and how these differences influence providers’ and clients’ outcomes. Better understanding of contextual variables could highlight barriers and facilitators to putting principles into practice, specific to different types of services in different political and economic contexts.

In further analyses of this rich data set, we intend to examine the roles of empowering community settings [28] features such as core activities, relational environment, roles, leadership, and mechanisms for setting maintenance and change in providers’ experiences of delivering homeless services. The relation between these characteristics could show how the goals and principles are put into practice. Deepening the research on empowering community settings could show some specific organizational characteristics of homelessness services.

In relation to practice, our findings emphasize the importance of creating opportunities to clarify and share the principles within the service, and the importance of values in the selection process of the providers. It is interesting that nobody mentioned policies, working protocol, or meetings, which could be useful to clarify the goals and principles of the organization and then the corresponding activities and procedures.

Based on our findings, support in recognizing and sharing goals and principles would be very useful psychosocial supervision for the teams working with people experiencing homelessness, not only to review key principles but also as an opportunity to share feelings of frustration related to difficulties in reaching goals, a very common experience to service providers who support people with complex needs [26].

## 5. Conclusions

Our research identified service providers’ perspectives about the goals and principles of their services at a cross-national level. Nevertheless, the research has some limitations. First of all, the small sample size (two or three teams for each countries) and the lack of specific criteria for the selection of the teams. To address this limitation, we recruited four service providers per FG, and eligible participants had at least six months of experience in the service.

Second, the researchers were different for each country and the FGs may have been influenced by the style of conducting of each one. To reduce this bias, the first authors of this paper developed a detailed protocol about planning (aims, recruitment, setting, role of the moderators and assistant, ethics), discussion (introduction, questions, conclusion, briefing), and content analysis.

Moreover, the final results were not analyzed by the researchers that conducted the FGs but by two independent researchers of the Italian team. To reduce potential bias and to enhance the trustworthiness of the interpretation, two strategies were used [37]: independent coders were appointed, and group discussions took place within the research team.

Third, the discussions were translated into English, and this could have affected the meaning of the questions and contents of participants’ responses. All the FG questions were translated into native languages and responses were then translated into English. However, the translation was necessary to allow for collaboration between consortium partners of different nations. Seven different languages were represented in this study. To reduce translation problems all partners used standardized translation/back-translation procedures [38] and when any doubts regarding translation arose, these were discussed among the consortium.

Despite these limitations, this is the first international study of the perspectives of homeless services providers. In the homelessness literature, the focus is usually on clients, not service providers, even though they are key for the success of homeless services and they are responsible for addressing one of the most complex expressions of poverty in modern society. With this research, we had the opportunity to analyze how providers conceptualize the goals and principles of their services that directly influence them, their work performance, and clients’ outcomes. Moreover, the trans-national perspective allowed us the opportunity to ‘fill in’ missing pieces in our knowledge of the ecology of homelessness. We hope this study encourages further research on service providers’ perspectives, and their relationship with the success of homeless services.

## Figures and Tables

**Table 1:** Goals of providers working with people experiencing homelessness.

Goals	Definition	Extracts
Support	Helping people achieving goals to improve their conditions	“The final aim of the professionals is to provide support to the user and to her/his situation, that can be very diverse and also imply different areas.” “Another aim at the institutional level is to bring people closer to the homelessness support network.” “To offer a personalized support to the person in order that everyone can set his/her objective.” “That they [the users] can achieve some sense of autonomy, feeling that they can control their own lives, that they have control.”
Integration	Helping people to feel more integrated in the society, as citizens and with a social support	“Have a team that helps people in this process of integration in the community, with the necessary for the support networks.” “Getting ties and networks in the community basically, trying to give them the support and aspects of support that they need to try and integrate back, and function in the community.” “That people access to the normalized network (…) as any other citizen, not only of the specialized network for homeless people.”
Basic needs	Satisfying the basic needs for survival (food, shower, safety, clothes)	“Those who moved in here, they will have a more structured life, support in abstinence and roof over their heads, and food.” “It’s actually warmth, food, a bit of company, you know? It’s really that baseline.” “Give him a meal, get dressed, be able to wash, that is, what they could not do before when they were on the street.”
Housing	Providing housing or finding accommodation for people experiencing homelessness	“To guarantee a worthy housing to the person who takes part in the program, that is his/her house and that is stable housing.” “The ultimate goal is that a person should go from homeless to non-homeless. That is, get a more permanent accommodation or lasting accommodation.” “It is necessary to work so that they can get back a dwelling in time.”
Well-being	Helping the people to improve their physical and psychological health	“The organization is ‘housing, health, recovery’.” “Health, with regard to addiction problems, which naturally also involves many risks.” “Allow people to get off the street if they wish, and to start on a path to recovery.”
Job/activities	Helping people to find a job or activities to have financial resources or something to do during the day	“Bringing finances in order and having something to do during the day.” “Finances, of course, are then important to maintain that house if they already have that house.” “Not only the house, they need to work but the job is a bit difficult for everyone, however, it is to incorporate them into society, making them feel useful and appreciated.”

**Table 2:** Principles of providers working with people experiencing homelessness.

Principles	Definition	Extracts
Dignity, respect, humanity	Working with people without prejudice, giving them respect and dignity, listening to them without judgment.	“Humanism, activism, benevolence, patience, selflessness.” “Just treating people with respect where they might have never got it before that they will get it off us when they come in the door.” “One of the principles is to estimate the person for the value she/he has.” “Treating people with respect, dignity, (...), giving them a voice and giving people a chance to change.”
Help everyone	Help all people in need, without constraints or access limits. Try to give everyone a chance to change, regardless of the person’s problems.	“[name organization] has a vision that they are there for everybody and that they are not selective in attracting certain clients just for financial benefit.” “No matter how many problems you have, you will be helped.” “In general, the [services] always welcomes everyone, whoever arrives, without prejudices.”
Person-centered approach	Put the person, her/his choices, path and aims at the center of the support.	“The main value is to allow our patients to self-identify.” “The intervention is very individualized, that is, being very person-oriented and being appropriate to the person with whom we are dealing.” “Our actions plan is very much person-centered.”
Housing as a right	Considering the home as the right of all people, trying to find a housing solution first of all.	“The house is a right and the house is where you will be, independently of how you decide to live.” “We wouldn’t be here if we didn’t have a strong belief that someone deserves a home.” “The home is fundamental.”
Social justice	Working for social justice and equality, believing that all people deserve social redemption regardless of their condition of life.	“We all have chosen to do this job because we really think that nobody should be excluded from society.” “Trying to give a chance to those who did not have it, just a sense of social justice.” “You work on the basis of equality. In regular care you usually have a client care relationship, we are trying to be much more equivalent on the level of the client. And I think that’s a big difference for people.”

**Table 3:** Organizational factors relating to the principles of providers working with people experiencing homelessness.

Organizational Factors	Definition	Extracts
Difficulty to put principles in practice	Difficulty in putting the principles into practice, due to organizational limitations (e.g., lack of resources, workload, difficulty in communication)	“[name organization] has a vision that they are there for everybody and that they are not selective in attracting certain clients just for financial benefit. Maybe, this is the main problem: the financial aspect. But this organization is indeed available for everybody.” “When you have a 100–115 cases workload, (…), you cannot support each person as a professional as he/she deserves.” “I understand that we put the persons at the center, but on the other hand it is just what is written. On our daily work we find some pressures (by the coordinator) that make the service leave people aside.”
Importance of having a mission	Importance of having a mission that guides the daily work, despite the difficulties	“I started off as a volunteer and doing it for free for a long time [laughs] so there has to be something that attracts you and for me it was the values. We’re not going to change the world, or throw it in your face, but we are going to stick with it and try and get the best outcomes possible.” “I think the written values facilitate the work.”
Shared/Not shared principles	Sharing values with colleagues and having a united team	“We can work very differently but I think our vision is always the same. We all have a common goal and a common mission.” “In the organization. I think we all have a common goal. Now obviously we work with a lot of different services across all of the midlands which may not have the same goals as us but in, we have a good ethos and we want to help people.” “It is not just sending an e-mail saying: ‘these are the new values, principles or mission’, but being able to work them together.”
Multidisciplinary	Sharing the responsibility among the staff members, with different professionals, and adopting a broader approach	“Multi-responsibility also allows us to regulate emotional impacts.” “I think we are quite flexible. Like when there is a change like that, do you know, because we work well, well as team like do you know, we manage it.”
Create innovation	Sharing the principles of the services with partners outside the team, to create innovation in the service delivery	“So it can be hard because we can’t necessarily challenge somebody else’s opinion or values, but if we had that control over that, if the team was all the same, if the people who provide accommodation were all on the one page, it would be different.” “Linking practice and politics and trying to somehow move between how the practice of social intervention from a more innovative perspective can contribute to changing social policies.”

**Table 4:** Service’s goals emerged within HF and TS programs in eight European countries.

Goals	Subthemes	Number of Services per Country ^1^																No. HF	No. TS	No. Countries
Total services involved		France		Ireland		Italy		Netherlands		Poland		Portugal		Spain		Sweden				
		HF	TS	HF	TS	HF	TS	HF	TS	HF	TS	HF	TS	HF	TS	HF	TS			
		2	2	3	2	2	2	2	2	-	2	2	-	2	2	2	2			
Support	Support individualized needs		1	2	1	2	2				2				2			4	8	5
	People decide the goals	1	1	1		1		2						2				7	1	5
	Connect to services			1	1							1			2			2	3	3
	To get out of homelessness	1		1	1						1	1		1	2		1	4	5	6
	Autonomy	2				2						1		2		2		9	-	5
Integration	Social network			1		1	1					1					1	3	2	4
	Community integration	1	1	1			2					2		2	2			6	5	5
Basic needs	Food, shower, clothes	1		1			2					1			1	1	1	4	4	6
	Safety			1			1		2									1	3	3
Housing	Give a house	1		2		1		2	1			1		2		2		11	1	7
	Find a house						2		1								2	-	5	3
	Temporary accommodation		2		1				1		1						1	-	6	5
Well-being	Health	1	2	1			1		1						1		2	2	7	6
	General well-being	1		1			1	1								1		4	1	5
Job/activities							1		2						1		1	-	5	4

^1^ The tables show the number of services in which each goal/principle emerged. The empty spaces indicated the absence of discussion about that principle.

**Table 5:** Service’s principles emerged within HF and TS programs in eight European countries.

Levels	Principles	Number of Services per Country ^1^																No. HF	No. TS	No. Countries
Total services involved		France		Ireland		Italy		Netherlands		Poland		Portugal		Spain		Sweden				
		HF	TS	HF	TS	HF	TS	HF	TS	HF	TS	HF	TS	HF	TS	HF	TS			
		2	2	3	2	2	2	2	2	-	2	2	-	2	2	2	2			
Principles guiding the relationship with clients	Dignity, respect, humanity	1		2	2	1	1				1	2						6	4	5
	Help everyone			2	2	1	1	1	2		2							4	7	4
	Person-centered approach	1		2		1		1	1		1	2		1	2	1		9	4	8
	Housing as a right			3		2						2		1		1		9	-	5
	Social justice					1	1	1	1			1						3	2	3
Organizational factors relating to the principles	Difficulty to put principles in practice			2	2	1		1	2					2	1			6	5	4
	Importance of having a mission			2		1					1	1		1	1			5	2	5
	Shared/Not shared principles	2		2	1	1	1				1	1		2	2	2		10	5	7
	Multidisciplinary	2			1							1						3	1	3
	Create innovation			1		1						1						3	-	3

^1^ The tables show the number of services in which each goal/principle emerged. The empty spaces indicated the absence of discussion about that principle.

## Data Availability

The datasets generated and/or analyzed during the current study will not be publicly available due to restrictions imposed by internal property rights of the HOME-EU Consortium study group but will be available from the corresponding author upon reasonable request.

## HOME-EU (Horizon 2020 GA/726997) Consortium Study Group

José Ornelas (Principal Investigator), Maria J. Vargas-Moniz, Maria F. Jorge-Monteiro (APPsyCI—Applied Psychology Research Center Capabilities and Inclusion, ISPA-Instituto Universitário, Rua Jardim do Tabaco, 34, 1149-041 Lisboa, Portugal); Ronni Michelle Greenwood, Rachel M. Manning, Branagh O’Shaughnessy (Department of Psychology, University of Limerick, Limerick, V94 T9PX, Ireland); Inês Almas, Teresa Duarte (AEIPS–The Association for Study and Psychosocial Integration; Housing First project: Casas Primeiro, Av. António José de Almeida, 26, 1000-043 Lisboa, Portugal); Francesca Disperati, Marta Gaboardi, Michela Lenzi, Massimo Santinello, Alessio Vieno (Department of Developmental and Social Psychology, University of Padova, Via Venezia 8, 35131 Padova, Italy); Rita P. Marques, Maria Carmona, Américo Nave (Crescer–Community Intervention Association, Bairro Qta Cabrinha 3–E/F 1300-906 Lisboa, Portugal); Freek Spinnewijn (FEANTSA, European Federation of National Organisations Working with the Homeless, Chausse de Louvain 194 Bruxelles 1210, Belgique); Roberto Bernad, Borja Rivero, Martin Julián (Rais Fundación, C/Ardemans 42, 28028 Madrid, Spain); Anna Bokszczanin, Barbara Zmaczynska–Witek, Skałacka Katarzyna, Aleksandra Rogowska (Institute of Psychology, Opole University, Pl. Staszica 1, 45-052 Opole, Poland); Sandra Schel, Yvonne Peters, Tessa van Loenen, Liselotte Raben, Judith R. Wolf (Radboud university medical center, Radboud Institute for Health Sciences, Impuls—Netherlands Center for Social Care Research, Geert Grooteplein 27, 6525 EZ, Nijmegen, The Netherlands); Ulla Beijer, Mats Blid, Hakan Kallmen (STAD, Stockholm Center for Psychiatry Research and Education, Karolinska Institutet, Norra Stationsgatan 69, 113 64 Stockholm, Sweden); Teresa Bispo, Tiago Cruz, Carla Pereira (Câmara Municipal de Lisboa [The Lisboa City Council], Praça do Município 38, 1100-038 Lisboa, Portugal); Pascal Auquier, Junie M. Petit, Owen Taylor (Aix-Marseille Univ, School of medicine–La timone Medical Campus, EA3279 CEReSS–Health Service Research and Quality of Life Center, 27 Boulevard Jean Moulin, 13385 Marseille, France), Sandrine Loubière, Aurélie Tinland (Department of Research and Innovation, Support Unit for clinical research and economic evaluation, Assistance Publique–Hôpitaux de Marseille, 27 Boulevard Jean Moulin, 13385 Marseille, France).
